# Effects of Interfaces of Goethite and Humic Acid-Goethite Complex on Microbial Degradation of Methyl Parathion

**DOI:** 10.3389/fmicb.2018.01748

**Published:** 2018-08-03

**Authors:** Gang Zhao, Enze Li, Jianjun Li, Meiying Xu, Qiaoyun Huang, Xingmin Rong

**Affiliations:** ^1^Guangdong Provincial Key Laboratory of Microbial Culture Collection and Application, Guangdong Institute of Microbiology, Guangzhou, China; ^2^State Key Laboratory of Applied Microbiology Southern China, Guangzhou, China; ^3^College of Resources and Environment, Huazhong Agricultural University, Wuhan, China

**Keywords:** ATR-FTIR, goethite, HA-goethite complex, microbial degradation, mineral interface

## Abstract

Microbial degradation plays an essential role in the removal of hydrophobic organic compounds (HOCs) dispersed in soil and sediment, and its performance is greatly affected by mineral particles which regulate HOCs bioavailability by interfacial adsorption. Likewise, bacteria cells attach to the surfaces of mineral particles as well but how bacterial attachment affects biodegradation is largely unknown. Here we report inhibitory effects of goethite and humic acid (HA)-goethite complex addition on microbial degradation of methyl parathion (MP). Using attenuated total reflectance-Fourier transform infrared spectroscopy, we observed that the adhesion of bacterial cells responsible for MP degradation on goethite occurred and the adhesive strength increased over time. We then replaced goethite with phosphate-adsorbed goethite to weaken the goethite-bacteria association and the inhibition of MP biodegradation was alleviated. These results suggested the formation of goethite-bacteria association hinder MP biodegradation. Meanwhile, our results showed that HA coating prevented bacterial attachment on goethite particles along with a drastically increased MP adsorption by goethite. The combined effect would lead to decreased mass fluxes of MP to bacterial cells and could represent another mechanism responsible for the decreased degradation rate observed in the current study.

## Introduction

The occurrence and fate of hydrophobic organic compounds (HOCs) such as polycyclic aromatic hydrocarbons and hydrophobic organic pesticides in the environment have received increasing attention because they are potentially toxic, carcinogenic, and persistent to degradation. Microbial degradation plays a key role in the attenuation of HOCs, and it greatly regulated by solid particles in sediment and soil because of HOCs adsorption on particle surfaces (Cycoń and Piotrowska-Seget, 2016; Cycoń et al., 2017; Mansouri et al., 2017; Ren et al., 2017). Generally, sorbed organic compounds are considered unavailable for microbial degradation without prior desorption (Bouchez et al., 1995; Theng et al., 2001; Zhu et al., 2016). Bosma et al. (1997) suggested that only freely dissolved organic compounds can be taken up by bacterial cells. Thus the humin-bound polycyclic aromatic hydrocarbons become unavailable to microorganisms, and the low freely dissolved concentration in the presence of absorptive matrix would lead to slow biodegradation. However, some studies have contradictory findings. The presence of sediment particles was found to boost the biodegradation of chrysene and benzo[α]pyrene (Xia and Wang, 2008), and the presence of HA which adsorbed phenanthrene led to an instant increase in its biodegradation rate (Smith et al., 2009). These contradictory results highlighted the complexity of combined studies with solid particles and HOCs, but the role of solid particles in the degradation of HOCs by microorganisms remains uncertain.

In the environment, soil/sediment solid particles not only adsorb organic pollutants but also provide a surface for microorganisms. An increasing body of literature indicates that bacterial attachment to the surfaces of solid particles could also significantly influence such microbial degradation. For instance, bacterial adhesion to activated carbon particles seemed to improve phenanthrene diffusion to bacterial cells, thus facilitating its biodegradation (Leglize et al., 2008). Zhang et al. (2012) found that the fraction of biodegraded humin-bound phenanthrene was significantly higher than that of the desorbed phenanthrene. They proposed that attached bacteria could consume phenanthrene on the humin. However, despite the potential importance of bacteria-particle interactions in microbial degradation, the experimental evidence failed to distinguish the contribution of microbial behaviors brought by planktonic and attached bacterial cells in the presence of solid particles like clay, which are similar in size to bacterial cells. This constraint has hindered us from determining the importance of the interactions between degrading cells and small mineral particles during biodegradation. The contribution of bacterial adhesion on mineral particles in microbial degradation of organic pollutants remains unclear.

The adhesion of microorganisms on solid particles is ubiquitous in soil and sediment environments. Once in the environment, bacteria tend to aggregate on the surface of Fe (hydr)oxide minerals because of their opposite surface charges in most circumstances (Chenu and Stotzky, 2002). Our previous research found that 92.3% of *Pseudomonas putida* cells bound to the mineral particles when growing with goethite (Rong et al., 2010). On the other hand, the bare surfaces of Fe (hydr)oxides are competed not only by bacteria but by other reactive substances like humic acid (HA) too. The sorption of HA to Fe (hydr)oxide surfaces can change the mineral surface properties, thus affecting their interaction with bacterial cells (Johnson and Logan, 1996; Ouyang et al., 2018). In addition, the sorption of HA to Fe (hydr)oxide surfaces can enhance the sorptive interactions for HOCs (Murphy et al., 1992, 1994). It is thus attractive to explore the different mechanisms in microbial degradation before and after adsorption of HA on Fe (hydr)oxides.

This study aimed to explore the role of goethite and HA-goethite complex in affecting biodegradation by analyzing the interface behavior of methyl parathion (MP) and bacterial cells. Goethite was selected as model particle due to its wide distribution in surface waters, soils, sediments, and other natural environments (Cornell and Schwertmann, 2003). HA is ubiquitously present in environments and has been reported to interact with Fe (hydr)oxides (Weng et al., 2006, 2007). MP (O,O-dimethyl O-p-nitrophenyl phosphorothioate), a hydrophobic organophosphorus pesticide, was chosen because of its wide application on nearly 70 different crops in several countries (Ragnarsdottir, 2000). The biodegradation kinetics of MP were compared in the presence of goethite or HA-goethite complex. To analyze the *in situ* bacterial behavior on the mineral surface in the degradation system, attenuated total reflectance-Fourier transform infrared (ATR-FTIR) spectroscopy was used. The sorption of MP and microcalorimetric experiments were carried out to determine the spatial distribution of MP and to characterize the intrinsic activity of MP-degrading bacterial cells.

## Materials and Methods

### Chemicals and Minerals

Methyl parathion (>99%) was obtained from the National Suspecting and Testing Center for Pesticide Products, China. Goethite was synthesized according to Atkinson et al. (1967) and characterized by Powder X-ray diffraction. HA-goethite complex (∼3.4% w/w) was prepared similarly to our previous publication (Hong et al., 2015). Hydrodynamic diameters and zeta potentials of minerals and bacterial cells were analyzed by zeta potential analyzer (ZetaPlus, Brookhaven Instruments, United States). Specific surface area (SSA) of minerals were analyzed using N_2_ adsorption (Beijing Analytical Instrument Company, China).

### Bacterium and Growth Condition

The bacterium, *Pseudomonas* sp. Z1, capable of utilizing MP as the sole carbon source was used in this study (Zhao et al., 2014). *Pseudomonas* sp. Z1 was inoculated in a 100 mL minimal salt medium (MSM, NH_4_Cl 1 g L^-1^, FeSO_4_⋅7H_2_O 0.001 g L^-1^, NaCl 0.5 g L^-1^, CaCl_2_⋅2H_2_O 0.0296 g L^-1^, MnSO_4_⋅H_2_O 0.001 g L^-1^, MgSO_4_⋅7H_2_O 0.986 g L^-1^, Hepes 2.383 g L^-1^) containing MP (30 mg L^-1^) and yeast extract (200 mg L^-1^) at 28°C and 180 rpm for 10 h. Then, the cells were harvested by centrifugation at 6000 ×*g* for 10 min at 28°C and washed three times with MSM. Finally, the cells were resuspended in MSM for subsequent use.

### Adsorption Experiment

A batch of known amount of minerals and 4 mL MSM were added to flasks. Then stock solution of MP was spiked into the flasks to achieve final concentrations ranging from 0 to 50 mg L^-1^. Samples were shaken at 28°C, 180 rpm for 4 h in the dark and then centrifuged at 20,000 ×*g* for 10 min. The supernatant concentration of MP was measured by high-performance liquid chromatography (HPLC) (Zhao et al., 2014). All samples were conducted in triplicates. The concentration of MP adsorbed to mineral particles was calculated by the amount of MP added and the remaining in the supernatant.

### Biodegradation Rate and ATR-FTIR Measurements

An experimental system for simultaneously collecting infrared spectra and degradation data was designed to evaluate the effect of mineral particles on MP biodegradation. ATR-FTIR spectra were collected using a ZnSe crystal element in a horizontal ATR cell (Pike Technologies, Inc.) installed in a Bruker Vertex 70 FTIR Spectrometer. ATR-FTIR measurement was performed in a similar way to previous publications with minor modifications (Elzinga et al., 2012; Wei et al., 2016). Briefly, a mineral overlayer was deposited onto the ZnSe crystal at 75°C for 3 h under N_2_-atmosphere. Two glass chambers were connected to the ATR cell. One chamber was sealed with a rubber stopper, and a mechanical modulator driver was used to circulate suspension at a flow rate of 2 mL min^-1^. A volume of 50 mL bacterial suspension (about 10^7^ cells mL^-1^), mineral (20 g L^-1^), and MP (30 mg L^-1^) was pipetted into the chamber and pumped over the overlayer. At intervals of 2 h, spectra were recorded in the range from 1000 to 1800 cm^-1^ at a resolution of 4 cm^-1^. At the end of the experiment, the goethite/HA-goethite complex deposit was inspected for any signs of film erosion, which were not observed. A bank spectrum consisting of the combined absorbance of MSM, the mineral deposit, and ZnSe crystal was collected as the average of 256 scans with a 4 cm^-1^ resolution, and all successive spectra were subtracted from this background spectrum.

Meanwhile, degradation data were also collected. At predetermined times, the samples were taken out from the chamber and centrifuged. The supernatant solution from centrifugation was extracted by solid-extraction using Agela Cleanert C18, and the SPE cartridge was eluted with 20 mL methanol. The pellets were extracted with methanol for three times which recovered near 100% MP. The supernatant and extractions were concentrated to 2 mL and analyzed by HPLC. For assessing the abiotic loss of MP during degradation, uninoculated controls were prepared using the same procedure.

To determine whether the influence of goethite on MP biodegradation was due to the association of mineral particles with bacterial cells, we evaluated MP biodegradation in the presence of phosphate-adsorbed goethite in the same manner as above. Phosphate has been proved very effective to decrease the quality of bacterial cells attachment onto Fe (hydr)oxides (Appenzeller et al., 2002). Phosphate-adsorbed goethite was prepared in KH_2_PO_4_ at a concentration of 7 mM. Goethite (20 g L^-1^) was reacted with PO_4_^3-^ in MSM for 48 h and stored for subsequent experiments.

### Microcalorimetric Experiment

The metabolic power-time curve of *Pseudomonas* sp. Z1 in the absence or presence of goethite and HA-goethite complex was recorded by TAM III thermal activity monitor (TA Instrument, United States). The whole equipment was sterilized before the experiment. Experiments were performed at 28°C in 4 mL stainless steel ampoules with 2 mL MSM containing bacteria (about 10^7^ cells mL^-1^), MP (30 mg L^-1^), and mineral (40 mg). The ampoules were placed in the calorimeter and signals obtained during growth were recorded by a TAM assistant software. Dissolved HA did not yield any heat signal no matter in MSM, MP-free control, or bacteria-free control, which also indicated that *Pseudomonas* sp. Z1 could not use HA as a growth substrate. Therefore, the captured heat signal was caused by the metabolic activities of *Pseudomonas* sp. Z1.

The microbial growth rate constant (μ) was obtained from the equation (Zhang et al., 2006):

$${\text{In}P}_{t}={\text{In}P}_{0}+\mu t$$

where *P_0_* and *P_t_* were the heat output power of the bacterium at time 0 and *t* min, respectively. Using the data In*P_t_* and *t* taken from the curves to fit a linear equation, we obtained the metabolic rate constant (μ) of degrading bacteria.

## Results and Discussion

### Surface Characterization

The properties of bacterial cells and goethite/HA-goethite complex were measured with respect to zeta potential, hydrodynamic diameter, and N_2_ sorption (**Table 1**). The zeta potential of *Pseudomonas* sp. Z1 and goethite were -27.81 ± 0.62 and -6.93 ± 0.53 mV in MSM, respectively. Similar to the previous report, the zeta potential of HA-goethite complex decreased to -41.54 ± 0.83 mV, as a result of HA coating (Antelo et al., 2007).

**Table 1:** Zeta potential, SSA, and size of bacteria cell and minerals.

Particle	Zeta potential (mV)	SSA (m^2^ g^-1^)	Size distribution mean (nm)
Strain Z1	-27.81 ± 0.62	–	1352
Goethite	-6.93 ± 0.53	50.71 ± 2.55	212
HA-Goethite complex	-41.54 ± 0.83	50.09 ± 3.27	298

*The zeta potential and particle size were analyzed at pH 7.0, 28°C. Data were presented by value ± standard deviation.*

### Adsorption Behavior of MP

**FIGURE 1:**
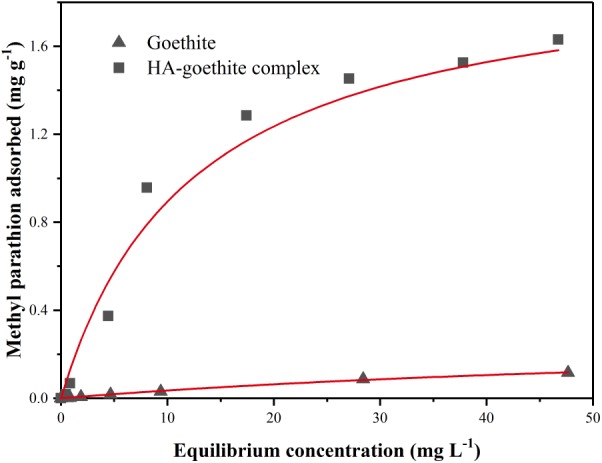
Sorption curves of methyl parathion (MP) on goethite and HA-goethite complex.

The equilibrium adhesion isotherms of MP to goethite and HA-goethite complex in MSM were shown in **Figure 1**. The adsorption data conformed to the Langmuir equation (Zhao et al., 2014). The maximum amount of MP adsorbed on the HA-goethite complex was 6.7 times higher than that on goethite. HA is an extraordinarily active soil fraction of HOCs, and organophosphate insecticides may bind to soil humic substances covalently (Parris, 1980). The sorption values of pesticides on soil organic matter are several times greater in extent than that of the mineral constituents (Almendros, 1995).

### Microbial Degradation of MP

**FIGURE 2:**
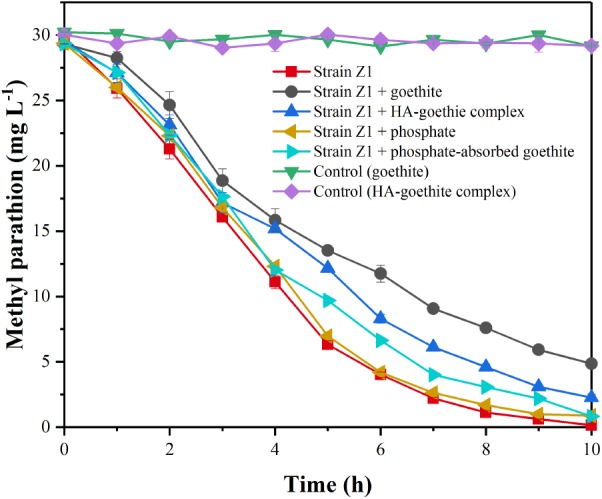
Effect of minerals on biodegradation of MP by *Pseudomonas* sp. Z1.

The biodegradation of MP was evaluated by comparing the extents and rates of degradation in the mineral-free against mineral-containing systems. No MP loss was found in abiotic processes. In the mineral-free system, 86.57 ± 0.65% of MP was degraded within 6 h and a 100% degradation was reached within 10 h. When goethite and HA-goethite complex were present, MP degradation dropped to 60.84 ± 2.18% and 72.34 ± 1.09% (6 h, **Figure 2**). The first-order rate constant for MP degradation was 0.16 ± 0.03 h^-1^ in the absence of mineral, and decreased to 0.10 ± 0.02 h^-1^ and 0.11 ± 0.02 h^-1^, respectively, in the presence of goethite and HA-goethite complex, suggesting an inhibitory effect on the biodegradation of MP.

To verify whether the inhibition of goethite on MP biodegradation was caused by the association of mineral particles with bacterial cells, the MP biodegradation in the presence of phosphate-adsorbed goethite were evaluated. Using density gradient separation (Jiang et al., 2007), we separated unattached bacteria fraction from bacteria-mineral systems. The result showed that the attached bacterial cells decreased by 78.86 ± 3.85% in phosphate-adsorbed goethite system compared to the non-phosphate treatments, suggesting that phosphate could effectively reduce the amount of strain Z1 attached on the surface of goethite (Supplementary Figure S1). As shown in **Figure 2**, the first-order rate constant for MP degradation in the presence of PO_4_^3-^ (0.16 ± 0.03 h^-1^) was very close to that of non-phosphonate treatments (0.15 ± 0.03 h^-1^) and no significant difference between them (*p* > 0.05), suggesting that phosphate itself would not introduce bias. For phosphate-adsorbed goethite system, MP degradation reached 97.21 ± 0.65% within 10 h, which was significantly higher than those observed under non-phosphate treatments (*p* < 0.01), suggesting an increase of degradation rate with decreasing bacterial adhesion quantity.

### Online *in Situ* ATR-FTIR Analysis

**FIGURE 3:**
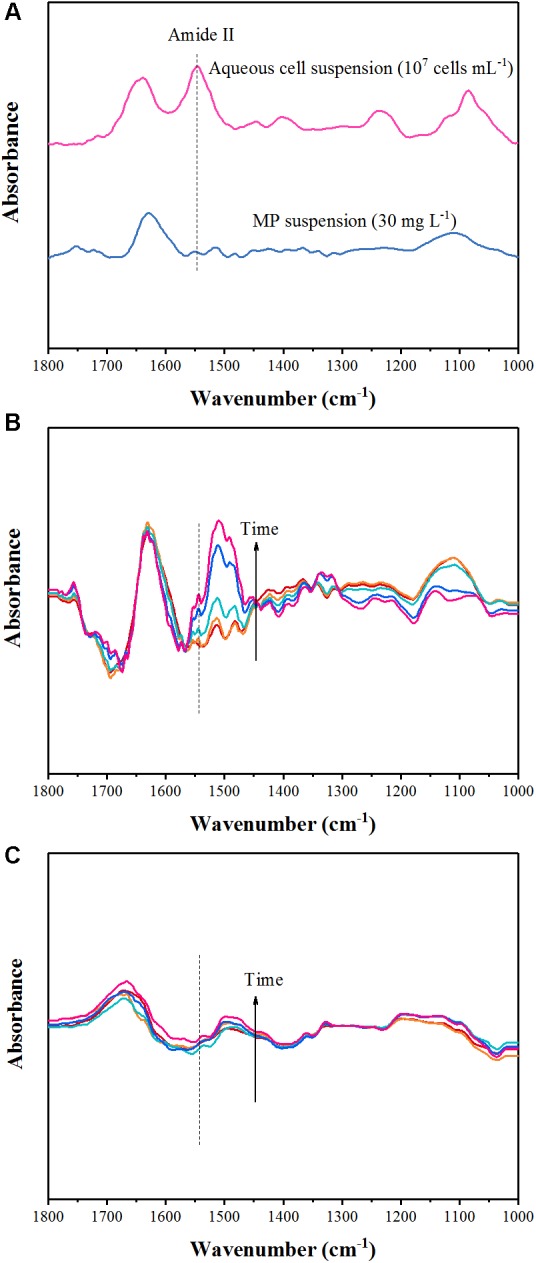
Attenuated total reflectance-Fourier transform infrared (ATR-FTIR) spectra of aqueous *Pseudomonas* sp. Z1 suspension and MP solution **(A)**, and collected as a function of time from the biodegradation experiment of the reaction between *Pseudomonas* sp. Z1 and goethite **(B)** and HA-goethite complex **(C)**.

**Figure 3A** showed ATR-FTIR spectra of *Pseudomonas* sp. Z1 cells (10^7^ cells mL^-1^) and MP (30 mg L^-1^), collected by using ZnSe ATR crystal in contact with suspensions. The adsorption bands of bacterial cell spectrum in 1800–1000 cm^-1^ region were consistent with previous ATR-FTIR studies characterizing bacterial strains (Parikh and Chorover, 2006; Elzinga et al., 2012). The spectrum of bacterial cells showed the main absorption bands at ∼1644 and ∼1546 cm^-1^ due to the vibrations of amide I and amide II of proteins; ∼1406 cm^-1^ caused by symmetric stretching of COO^-^; ∼1237 cm^-1^ related to asymmetric stretching vibration of PO^2-^; and ∼1085 cm^-1^ due to vibrations of C–OH, P=O, and P–O) (Parikh and Chorover, 2006). We chose the signal at ∼1,546 cm^-1^ to represent the bacterial coverage because the amide I band was often perturbed by water-related adsorptions and the amide II spectrum was the least affected (Shephard et al., 2010). The ATR-FTIR spectrum of MP contained several adsorption bands, which located at 1630, 1514, 1481, 1366, 1109, 1082, and 1036 cm^-1^ in the range of 1800–1000 cm^-1^ (**Figure 3A**). The band centered around 1630 cm^-1^ usually appears with the IR absorption of water and the band at 1481 cm^-1^ is characteristic of υ_s_ (C=C) of the benzene ring (Movasaghi et al., 2008). Several adsorption bands including 1112 [υ (P-O-C) or υ (P=O)] and 1086 [υ_s_ (PO_2_^-^)] were found in the range of P-O vibrations of phosphonate (1200–1000 cm^-1^) (Movasaghi et al., 2008). ATR-FTIR experiments were well fitted the study of the degradation system, as the strong amide II band (1546 cm^-1^) characteristic of proteins was separated from the main IR bands of MP (**Figure 3A**). Hence, the IR spectroscopy was used to quantify total protein and bacteria absorbance, and interactions between degrading bacteria and mineral can be monitored in real-time.

For the goethite and HA-goethite complex systems, peak intensities declined compared with the spectrum of mineral-free bacterial cells on ZnSe, probably owing to the presences of mineral suspensions (**Figures 3B,C**). Compared to the HA-goethite complex system, more adsorption bands were observed in the range of 1800–1000 cm^-1^ in the goethite system, which due likely to the formation of chemical bonds between the catabolic intermediates of MP (i.e., paraoxon, dimethylthiophosphoric acid, *p*-nitrophenol, 4-nitrocatechol, and 1,2,4-benzenetriol) and Fe or hydroxyl groups on the surface of goethite (**Figures 3B,C**; Rani and Lalithakumari, 1994; Pakala et al., 2007; Tiwari et al., 2017a,b). For example, the emerging bands at 1106 and 1071 cm^-1^ were reported earlier from IR spectra of P adsorbed on Fe oxides (Tejedortejedor and Anderson, 1990; Hong et al., 2016).

The ATR-FTIR spectra of goethite system clearly revealed amide II band increased with time as the reaction progressed (**Figure 3B**). The data displayed that the adhesion of degrading bacterial cells to goethite occurred and enhanced over time under the conditions of degradation experiment. Our IR spectra depicting the attachment of *Pseudomonas* sp. Z1 to goethite were similar to the way how microbial cells adhered to hematite, as reported by Elzinga et al. (2012). Distinct changes in the amide II (1546 cm^-1^) range signaled the importance of surface proteins for bacterial attachment to the hematite surface. The strong interactions between bacterial cells and goethite may be attributed to electrostatic forces and bonding interactions such as inner-sphere complexation (e.g., inner-sphere P-OFe bond) and hydrogen bond (Rong et al., 2010; Wei et al., 2016). It is worth noting that the zeta potentials of goethite and *Pseudomonas* sp. Z1 are negative (**Table 1**), suggesting both surfaces are negatively charged. The zeta potential reflects the net or average electrokinetic properties of particles and the charge heterogeneities could result in preferential adhesion of bacterial cells on goethite (Walker et al., 2005). However, amide II was absent during the degradation experiment in the HA-goethite system (**Figure 3C**), indicating little attachment of degrading cells to HA-goethite complex. Hong et al. (2015) reported that the adhesion capacity of *Bacillus subtilis* decreased by 72% when the goethite surface was coated by HA. HA is negatively charged at neutral pH (Foppen et al., 2008). HA coating could deliver negative surface charges to goethite (**Table 1**) and subsequently inhibit bacterial adhesion.

### Metabolic Activity of *Pseudomonas* sp. Z1

**FIGURE 4:**
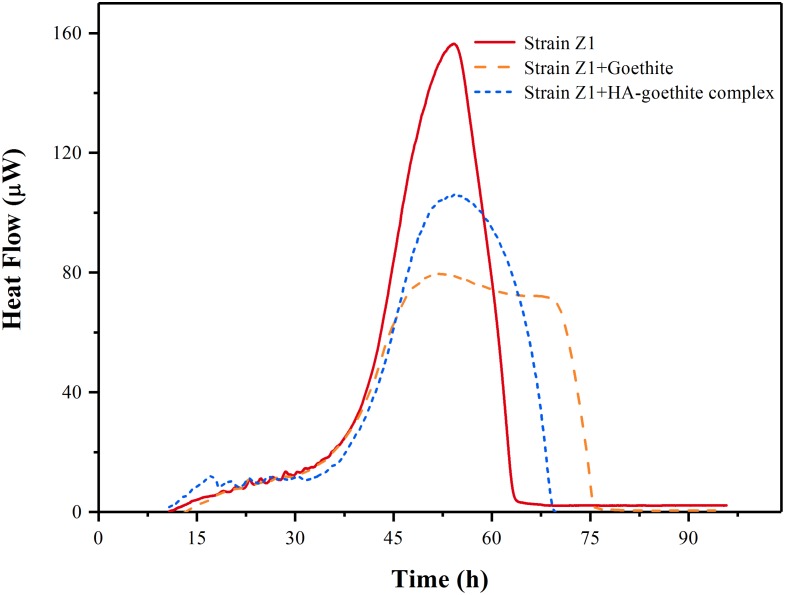
Calorimetric signal versus time for *Pseudomonas* sp. Z1 metabolism in the presence of goethite and HA-goethite complex.

Goethite and HA-goethite complex affected the thermal activity of strain Z1, as shown in **Figure 4**. We determined the microbial growth rate constant (μ) using microcalorimetry based on the assumption that the heat released from catabolic activities in the log phase is proportional to the rate of cell division (Zhang et al., 2006). Together with changes of the maximum heat flow (*P*_max_), we managed to identify whether the presence of goethite and HA-goethite complex was inhibitory or promotional in the bacterial activity. Highest μ values were observed under mineral-free treatment and decrease from HA-goethite complex to goethite system (**Table 2**). Bacterial metabolism exhibited a larger *P*_max_ (156.41 μW) under the mineral-free treatment, compared to the *P*_max_ under the HA-goethite complex treatment (105.71 μW), and the goethite treatment (79.47 μW) (**Figure 4** and **Table 2**). These results demonstrated that both goethite and HA-goethite complex depressed bacterial activity on MP degradation with stronger effect by goethite.

**Table 2:** Thermokinetic parameters of *Pseudomonas* sp. Z1 utilizing MP as a sole carbon source in mineral systems.

Systems	PT (h)	PH (μW)	μ (h^-1^)	*R*^2^
Strain Z1	54.20	156.41	0.154 ± 0.002	0.99
Strain Z1+ Goethite	52.35	79.47	0.127 ± 0.001	0.99
Strain Z1+ HA-Goethite	55.02	105.71	0.146 ± 0.001	0.98

*PT, peak time, when the calorimetric signal reaches the maximum; PH, peak heat, the maximum heat output detected; μ, the growth rate constant of Pseudomonas sp. Z1 in the log phase; R^*2*^, correlation coefficient used to judge the goodness-of-fit of a logarithmic growth model.*

### Influencing Mechanisms of Goethite and HA-Goethite Complex on the Biodegradation of MP

Our experiments showed that the formation of goethite-strain Z1 association is the critical factor in inhibiting biodegradation of MP. Our previous work found that goethite had a high affinity for bacterial cell and significantly inhibited the total metabolic activity and the sporulation of *Bacillus thuringiensis*. The tight binding of goethite on cells may hinder the uptake of substrates, and impair efflux of metabolites (Rong et al., 2007). Glasauer et al. (2001) observed that nanometer-scale Fe (hydr)oxides (ferrihydrite, goethite, and hematite) can be tightly attached to bacteria and suggested that, in some instances, the mineral crystals had even penetrated the outer membrane and peptidoglycan layers. Harms and Zehnder (1994) suggested that the attachment of *Sphingomonas* sp. strain HH19k to glass beads limited the uptake of dibenzofuran. It is possible that the association of strain Z1 with goethite would limit the diffusion of MP from aqueous phase toward bacterial cells, thus hinder biodegradation process.

In contrast, for HA-goethite complex system, most degrading bacterial cells were planktonic (**Figure 3C** and Supplementary Figure S1). It is reasonable to expect that MP biodegradation would not be inhibited by the presence of HA-goethite complex. Rather, the HA-goethite complex system as well resulted in a decreased rate of MP. We performed the MP adsorption experiment and found that instead of degrading bacteria, the HA-goethite complex had a high affinity for MP. Several studies found that the adsorption of HOCs on HA facilitated the microbial degradation process. In these studies, enhanced biodegradation was observed in the presence of humic fractions and they proposed that a fraction of HOCs associated with the solid particles be directly bioavailable to the attached bacterial cells (Poeton et al., 1999; Zhang et al., 2012). In this study, most bacterial cells were planktonic in the HA-goethite complex system. The different bacterial behaviors might relate to microbial species or experimental conditions. Therefore, the combined effect arising from the adsorption of MP by mineral particles and the weak association between degrading bacterial cells and HA-goethite complex could represent another mechanism responsible for the decreased degradation rate observed in the current study.

Further insight can be gained when considering that biodegradation of MP is also affected by the intrinsic cellular activity. The results of the microcalorimetric analysis showed that goethite and HA-goethite complex both inhibited the activity of degrading bacteria (**Figure 4**). Several mechanisms have been proposed for the loss in bacteria cell activity following interaction with Fe (hydr)oxides. Cai et al. (2013) reported that the viability of *E. coli* O157:H7 attached on goethite was initially ∼98% and then dropped to about 6% after 6 h. They deduced that the strong association of bacteria cells with goethite and the penetration of cell membrane by goethite caused cell death. Recently, Ouyang et al. (2018) demonstrated that hematite particles inhibited *Pseudomonas putida* growth mainly through elevated oxidative stress and physical interaction with cells. They also found surface-bound HA inhibited the adhesion to bacterial cells, thus alleviating the toxicity of hematite. In our study, decreased bacterial metabolic activity under the HA-goethite treatment might result from limited MP, sole carbon source, availability in the system as HA-coated goethite effectively absorbs much of the MP provided.

## Conclusion

In complex environments such as soil and sediment, it is difficult to clearly define the mechanism of processes that account for the roles of mineral particles in biodegradation of organic pollutants, particularly when physical, chemical, as well as biological factors are all simultaneously involved. Findings from this study suggest the surface of goethite and HA-goethite complex particles play an important role in affecting MP degradation by *Pseudomonas* sp. Z1. The goethite particles provide surface area for the attachment of degrading bacteria, and the surface of HA-goethite complex adsorbs MP. The interactions of mineral particles with degrading bacterial cells and MP would reduce substrate diffusion from the cell’s surroundings, and thus are crucial parameters for biodegradation. Further studies are needed before definitive conclusions can be made regarding the effects of the mineral surface on MP crossing the cell membrane. This work highlights the importance of the behaviors of contaminant and bacterial cells affecting microbial degradation of organic pollutants in soil and sediment.

## Author Contributions

GZ, MX, and XR designed the study and wrote the paper. GZ and EL operated the experiments. JL and QH discussed the results. All authors agreed to be accountable for the content of the work.

## Conflict of Interest Statement

The authors declare that the research was conducted in the absence of any commercial or financial relationships that could be construed as a potential conflict of interest.
