# Supplementary material for: Effects of Interfaces of Goethite and Humic Acid-Goethite Complex on Microbial Degradation of Methyl Parathion
Source: Front Microbiol. 2018 Aug 3;9:1748. doi: 10.3389/fmicb.2018.01748 (PMC6085511; doi:10.3389/fmicb.2018.01748)
Supplement: Supplementary file 1 [file Image_1.PDF]

Supplementary Material

Effects of Interfaces of Goethite and Humic Acid-goethite Complex on Microbial Degradation of Methyl Parathion

Gang Zhao [1, 2], Enze Li [1, 2], Jianjun Li [1, 2], Meiying Xu [1, 2*], Qiaoyun Huang [3], Xingmin Rong [3]**

[1] Guangdong Provincial Key Laboratory of Microbial Culture Collection and Application, Guangdong Institute of Microbiology, Guangzhou 510070, China

[2] State Key Laboratory of Applied Microbiology Southern China, Guangzhou 510070, China

[3] College of Resources and Environment, Huazhong Agricultural University, Wuhan 430070, China

*** Correspondence:**
Meiying Xu: xumy@gdim.cn.

Xingmin Rong: rongxm@mail.hzau.edu.cn

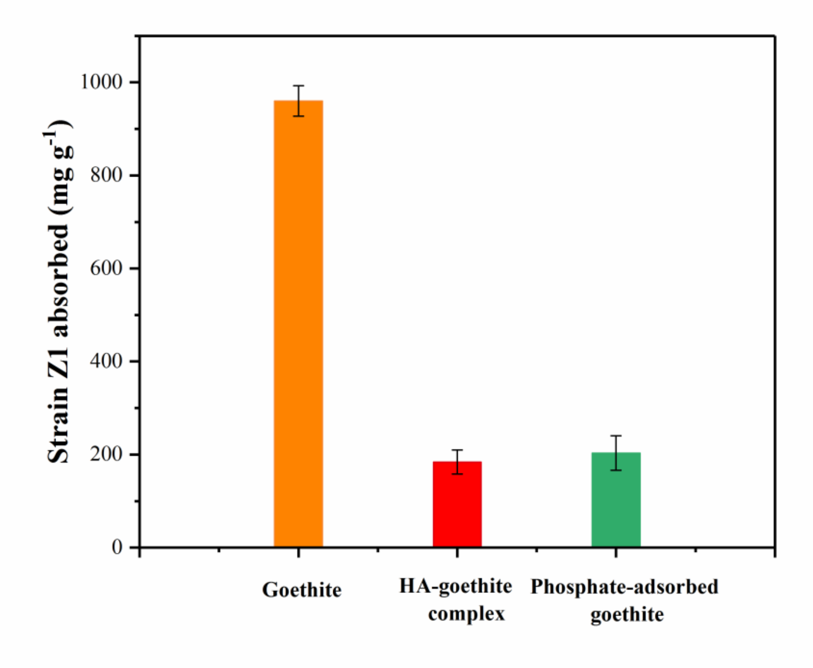

Supplementary Figure 1. Influence of surface-bound phosphate and HA on the adhesion of *Pseudomonas* sp. Z1. The quantity of adsorbed bacterial cells was evaluated using density gradient separation with minor modifications (Jiang, D., Huang, Q., Cai, P., Rong, X., Chen, W. (2007).

Adsorption of *Pseudomonas putida* on clay minerals and iron oxide. Colloids Surface. B 54, 217-221; Hong, Z.N., Rong, X.M., Cai, P., Dai, K., Liang, W., Chen, W., Huang, Q.Y. (2012). Initial adhesion of *Bacillus subtilis* on soil minerals as related to their surface properties. Eur. J. Soil Sci. 63, 457–466.). Briefly, 50 mg (dry weight) bacterial cells and 50 mg mineral were mixed with 30 mL MSM and shaken at 28 ^{o}C for 2 h. This high concentration of bacterial cells was used to clearly show the difference of bacterial adhesion on different surfaces. The free cells were separated by injecting a certain volume of sucrose solution (60 % by weight) into the bottom of the mixture and centrifuging. The quantity of free bacterial cells above the sucrose layer was analyzed using spectroscopy at 420 nm. The concentration of bacterial cells adsorbed to mineral particles was calculated by subtracting the amount of unattached weight from the initial weight.
